# 15 years of facts and figures on veterinary disciplinary measures in the Netherlands

**DOI:** 10.3389/fvets.2022.987797

**Published:** 2022-11-11

**Authors:** Iaira E. Boissevain, Myrthe van Rooij, A. W. Jongbloed, Franck L. B. Meijboom, Jan Willem Hesselink, Paul J. J. Mandigers

**Affiliations:** ^1^Department of Clinical Sciences, Faculty of Veterinary Medicine, Utrecht University, Utrecht, Netherlands; ^2^Division Animals in Science and Society, Department Population Health Sciences, Utrecht University, Utrecht, Netherlands; ^3^Molengraaff Institute for Private Law, Utrecht University School of Law, Utrecht, Netherlands

**Keywords:** complaints, disciplinary, measures, veterinary law, ruling

## Abstract

In most countries, a veterinary disciplinary system is in force to ensure the quality of the veterinary profession and to offer an objective platform for complaints. We present an analysis of 15 years of veterinary disciplinary verdicts (2001–2016) to compare facts and figures and identify which factors are of major influence on the outcome of the verdicts. Rulings were collected from both paper files and the digital database of the veterinary disciplinary council (VDC), categorized, and used to create a database that enabled a statistical analysis. The results showed that complaints pertaining to companion animals are filed predominantly by owners, whereas complaints about livestock are mostly filed by the governmental civil servant (CS). CS complaints mostly address compliance issues. For the complaints made by owners (client complaints, CCs), reporting, communication, and veterinary mistakes appeared to be of statistical significance. Further studies are needed to investigate the impact of the complaints on veterinarians in general and how we can improve the veterinary disciplinary system.

## Introduction

In most countries, like the Netherlands, a veterinary disciplinary system is in force to ensure the quality of the veterinary profession and to offer an objective platform for complaints. When first established in the Netherlands, its primary goal was animal welfare and food safety (1) but from the start, it served as a platform for client complaints (CCs) (2). Owners may perceive a veterinary disciplinary system as a body that they can use to file their complaints but it is actually a system to ensure quality and improve veterinary care (3, 4). Owners rarely realize the effect of their complaints. CCs have a huge impact on veterinarians and their co-workers (4–6). Recent studies investigating the effect of complaints in both human and veterinarian medicine have shown that complaints contribute to psychological distress such as depression, burnout, anxiety, and even suicidal ideation (7, 8), and health problems such as cardiovascular disease, insomnia, headaches, anger, and relationship problems (5, 9). Although the possibility of complaining may stimulate health workers to improve their level of care, the detrimental effect on job satisfaction and mental health (4–6) may warrant a critical analysis of these disciplinary systems as well.

The Dutch veterinary disciplinary system was first established in 1990. In contrast to the disciplinary system of, for example, the UK (10) or the USA (4), where complaints are first evaluated by a committee and only taken into consideration after careful scrutinizing, nearly all complaints sent to the Dutch veterinary disciplinary council (VDC) are taken into consideration. The original intent of the Dutch government, when establishing the VDC, had been the importance of veterinary care for livestock as it is one of the most important agricultural economic activities in Dutch (11). For this reason, the legal system was not designed to separate complaints in different ways. Regardless of the nature of the complaint, veterinarians feel forced to respond. This may have, like in other countries, a huge impact on their well-being (4–6). In the Netherlands, complaints can be filed either by a civil servant (CS) of the government or by an owner to the Dutch VDC (1, 12). Complaints raised by the CS often address compliance issues of general interest, whereas the CCs address many other issues. As written, the VDC addresses in principle all complaints unless the complaint is truly inadmissible. Complaints are inadmissible if filed by somebody who is not directly involved (for instance, a neighbor) or if they do not refer to veterinary care (for instance, a complaint referring to the costs of treatment). Complaints about communication, etc., are all addressed by the VDC as long as the complaint has some element that refers to the legal duty of care. If the CS, the client, or the owner is not satisfied with the VDC ruling, it is possible to appeal to the veterinary disciplinary appeal council (VAC). The only exception is the situation where the VDC considered the complaint found and issued disciplinary measures. The owner or CS cannot appeal for heavier measures.

The VDC consists of one lawyer, who is the chairperson, and four veterinarians. The VAC consists of three lawyers and two veterinarians. All members of the VDC and VAC are appointed by the Dutch Government (3).

Although VDC and VAC are in function for over 29 years, a scientific analysis of the nature of the complaints and the rulings of the VDC and VAC has not yet been performed. This study aimed to describe the sources of complaints, the reasons for the complaints, and the kind of rulings made.

## Materials and methods

For the analysis in this study, we chose the VDC rulings from the years 2001–2016. Rulings prior to this date were either incomplete or not available for this analysis and hence had to be excluded. At the time of analysis of this study, most of the rulings beyond this time frame were, for various reasons, not yet available to us and will therefore be analyzed in a different study.

This study was neither subjected to ethical approval as it does not involve any use or experimental use of animals nor will any data be disclosed that are subject to any privacy laws.

The rulings were systematically arranged in an inventory data set. The data were collected both from paper files and the digital database of the VDC. Only the data of complaints handled by the VDC and VAC are included in this Excel database.^1^ Complaints that have not been handled, or resolved for various reasons, such as administrative errors, have not been registered and are therefore unknown to us.

All rulings contained data that were used to establish different value labels which could be categorized into excel rows. Different value labels were created such as animal species, size, gender, age, type of complaint, outcome, etc. A complete list of these different value labels and their descriptions are found in Table 1. The value labels were coded into subcategories to enable further analysis. A complete list of these value labels and subcategories is listed in Appendices I and II.

**Table 1:** Value labels and coding used in the dataset veterinary disciplinary council (VDC) and veterinary disciplinary appeal council (VAC). The exact coding can be found in Appendices I and II.

**Value label**	**Description of value**	**Coding**
Date/number of the ruling	Publication date of the ruling and the number by which the ruling was published.	Year and number
Complainer	The person who filed the complaint	Owner or civil servant
Animal species	The animal species about which the complaint was filed	Horses, dogs, cats, rodents, farm animals, poultry, birds, and others
Animal age	Age group of the animal(s)	Younger than 1 year, 1–5 years, 5–10 years, 10–15 years, older than 15 years, various ages, unknown, and not applicable
Animal sex	The sex(es) of the animal(s)	Male, female, unknown, or inapplicable
Animal breed	Dog breed/size	Small, medium, or large dog and unknown breeds. Remaining animals and breed inapplicable
Animal deceased	Whether the animal was deceased at the moment the complaint was filed.	Yes, no, unknown, or inapplicable
Summary of ruling	Summary of the nature of the complaint	See Appendix 1 for further details of categories
Influence of reporting	Whether the accuracy of the patient record influenced the verdict	Yes or no
Outcome of ruling	Whether the ruling was founded or unfounded.	Unfounded, founded, dismissed, or inadmissible
Measure of ruling	Sentence which was given to the veterinarian	inadmissible (1) unfounded (2), warning or reprimand (3), fines (4), fine and/or suspension (5) and complete/partial removal of profession (6)
Appeal	Whether outcome of the ruling lead to appeal	Yes or no
Summary of appeal	Summary of the nature of the appeal	See Appendix II for further details of categories
Appellant	The person who filed the appeal	Veterinarian, owner, officer, or multiple parties
Outcome of appeal	The ruling of the Veterinary Disciplinary Appeal board.	Founded or unfounded
Measure of appeal	Sentence of the appeal different than the sentence of the disciplinary case	Lower, heavier or equal

The complete data set consisted of rulings of the VDC and VAC. The data sets were first checked for errors and missing values, and when possible, assigned a numeric property and imported into SPSS.^2^ All rulings, whether from the VDC or VAC, used the same value labels. The VDC rulings were grouped into six categories: (1) the complaint is either dismissed or inadmissible for instance due to administrative reasons, (2) unfound, (3) found with either no measure or a warning or reprimand, (4) a fine conditional or unconditional, (5) a fine and/or (un)conditional suspension, and (6) partial or complete removal of the veterinary profession. The VAC data are measured in equal, heavier, or lower rulings, and an additional parameter was the person who appealed to the ruling of the VDC, such as either the CS, veterinary, or the owner.

The veterinary disciplinary council data set was first analyzed using all rulings. First, the effect of time on the outcome was studied: found or unfound, and second, whether there was a time effect for the ruling itself. We used Kendall's tau association test because the rulings were non-parametric ordinal data.

As the complaints filed by the CS address mostly legal issues and hence are of a completely different nature compared to those filed by owners, the CS and CC rulings were analyzed separately. Each subcategory (Appendices I and II) was analyzed for the two types of rulings: CS and CC rulings. To see whether there was a statistically significant effect for each subcategory for either “found” or “unfound,” a Fisher Exact test with a *p*-value for the significance of <0.05 was used. The CS rulings were analyzed for (1) animal species, (2) status (deceased or not), (3) influence of reporting, and (4) the summary of the complaint. If the information was missing (for instance whether the animal had died or not), that ruling was excluded from the analysis.

The CC rulings were analyzed for (1) animal species, (2) sex, (3) age, (4) status (deceased or not), (5) influence of reporting, (6) dog breed, and (7) summary of the complaint. If the information was missing (for instance age or breed), that ruling was excluded from the analysis.

The veterinary disciplinary appeal council rulings were analyzed for (1) the person who appealed, (2) the type of appeal, and (3) the influence of reporting with as a dependent parameter the new ruling, using a Fisher Exact test with a *p*-value for significance <0.05. If applicable, a two-tailed Pearson correlation coefficient was calculated.

## Results

### Descriptive analysis rulings VDC

**Figure 1:**
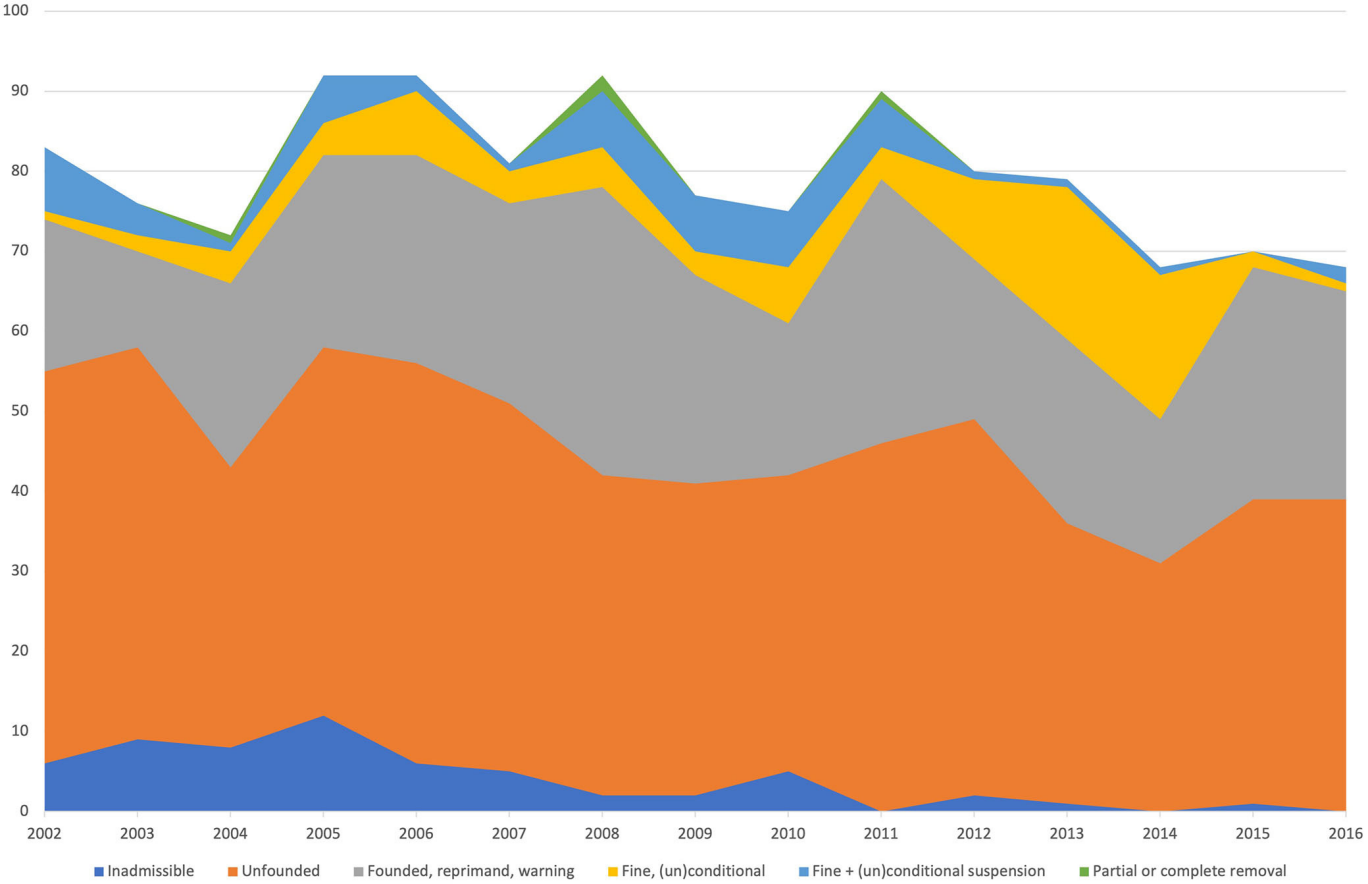
Rulings of the VCD for all cases filed from 2002 to 2016. The color corresponds with the ruling of the veterinary disciplinary council (VDC).

**Figure 2:**
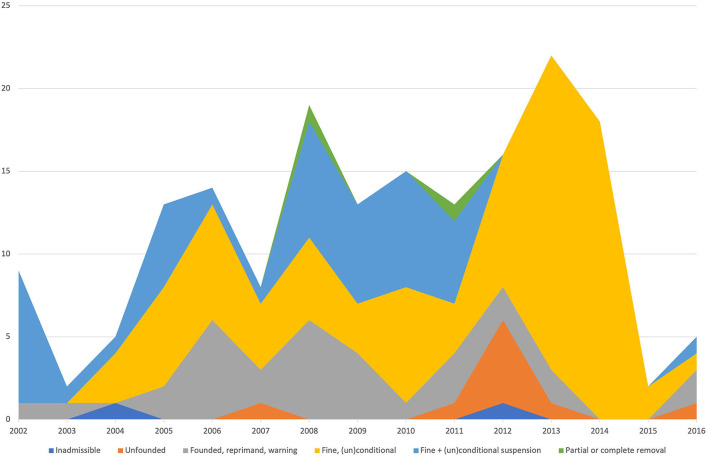
Rulings of the VDC for only cases filed by the civil servant (CS) from 2002 to 2016. The color corresponds with the ruling of the VDC.

**Figure 3:**
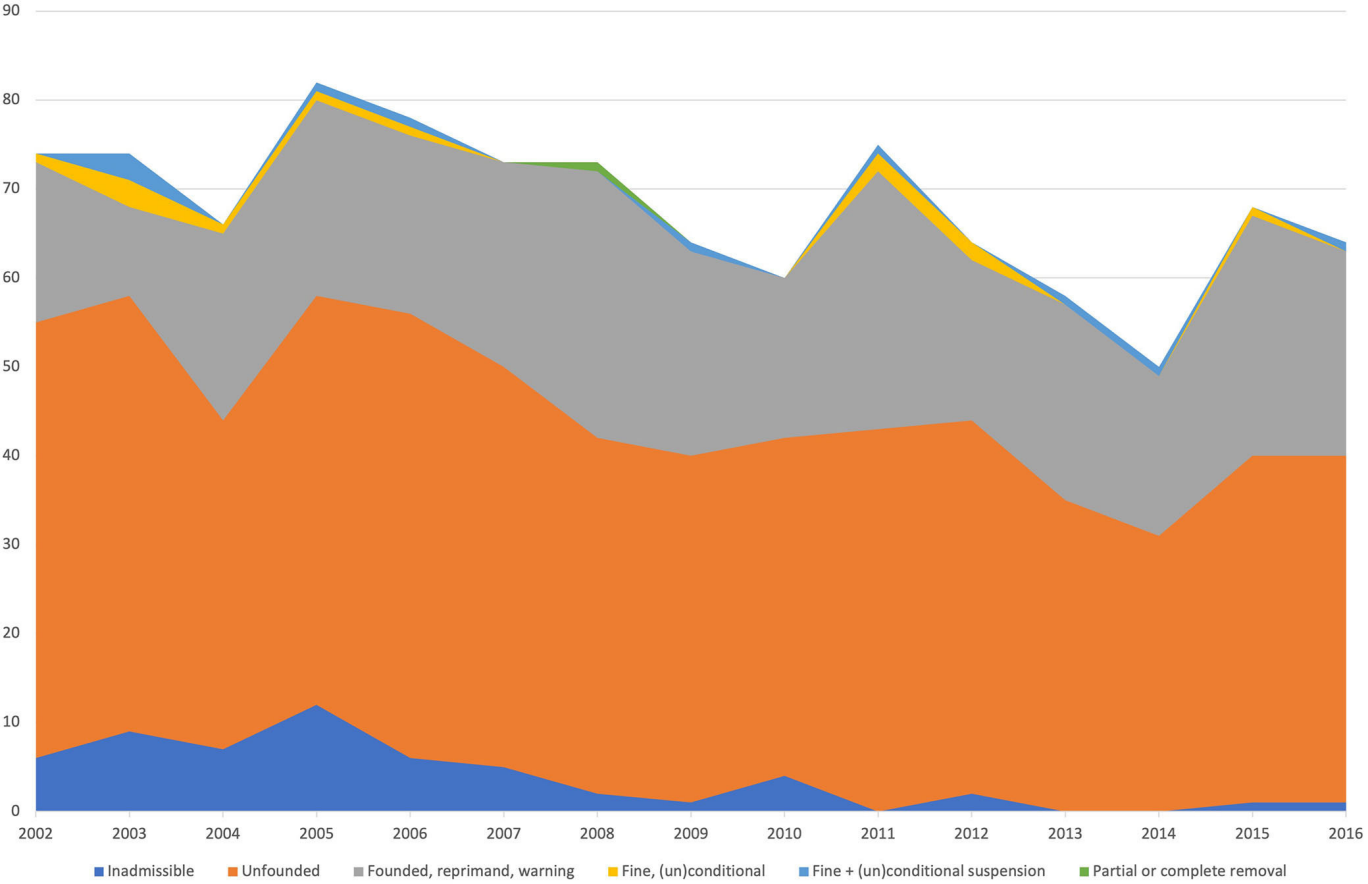
Rulings of the VDC for only cases filed from 2002 to 2016. The color corresponds with the ruling of the VDC.

A total of 1,197 verdicts were available for analysis. The number of complaints involving companion animals was 890 (74%) out of 1,197 verdicts; 157 (13%) complaints concerned equines, and 150 (13%) were related to livestock. In total, 1,023 complaints were filed by owners (CCs) (85%) representing a vast majority in contrast with the 174 cases filed by the CS (15%). The total number of cases did not vary greatly during this period. The mean number was 79 cases per year with a range of 68 to 92 (Figure 1). During the analyzed period, the number of CS cases gradually increased and the number of CC cases decreased. In both groups, the number of inadmissible cases declined during the analyzed period (Figures 2, 3).

Cases filed by the CS addressed more than 96% of compliance cases (Table 2). During the analyzed period, the number of CS cases gradually increased. During the period from 2008 to 2011, more rulings were made with (un)conditional fines and suspension (Figure 2). After this period, most rulings concerned fines (Figure 2), and this difference was statistically significant (*p* < 0.001). A similar statistically significant effect was observed for the year, irrespective of whether the ruling was found or unfound (*p* = 0.011). Cases filed in the period after 2008 were more likely to be found. The majority of CS cases concerned livestock (79%), 8% concerned companion animals, and 13% concerned equines (Table 2). There was no statistically significant difference for animal species, status, or influence of reporting, irrespective of whether the ruling was unfound or found (Table 2). However, there was a clear statistically significant difference if the summary of the case addressed compliance issues (*p* = 0.01).

**Table 2:** Descriptive statistic complaints of CS.

	**Categories**	**Cases unfounded (*n* = 9)**	**Cases founded (*n* = 165)**	***n* = 100%**	***p*-value**
Animal species	Companion animals Horses Livestock	1(7.1%) 2(8.7%) 6 (4.4%)	13 (92.9%) 21 (91.3%) 131 (95.6%)	14 23 137	0.429
Status	Deceased Not Deceased Unknown Not applicable	1 (4.5%) 3 (20.0%) 0 (0%) 5 (3.7%)	21 (95.5%) 12 (80%) 2 (100%) 130 (96.3%)	22 15 2 135	0.283
Reporting	Reporting important Reporting not important	8 (5.0%) 1 (6.7%)	151 (95.0%) 14 (93.3%)	159 15	0.565
Summary of the case	Compliance/general interest Duty of care	6 (3.7%) 3 (30%)	158 (96.3%) 7 (70.0%)	164 10	0.010

The number of CCs gradually decreased as mentioned above (Figure 3 and Table 4). This was partly due to the decline in inadmissible cases. There was a clear effect for year and ruling (*p* < 0.001). In the first analyzed period, the number of cases that were considered to be unfound decreased, whereas the number of cases that were considered to be found remained constant (Table 3). The CC cases were analyzed for several categories, irrespective of whether they were judged to be found or not. Animal species, sex, age, status (deceased or not), or dog breed had no statistically significant effect on the outcome of the ruling (Table 4). However, there was a clear statistically significant effect for reporting and the summary of the complaint (mistakes and communication) (Table 4). Poor reporting resulted more likely in a “found” ruling (*p* < 0.001). The statistically significant effect was even greater for communication and veterinary mistakes (*p* < 0.001).

**Table 3:** All client complaint (CC) cases per year and rulings.

**Year**	**Inadmissible**	**Unfounded**	**Founded, reprimand, warning**	**Fine, (un)conditional**	**Fine + (un)conditional suspension**	**Partial or complete removal**	**Total**
2002	6	49	18	1	0	0	74
2003	9	49	10	3	3	0	74
2004	7	37	21	1	0	0	66
2005	12	46	22	1	1	0	82
2006	6	50	20	1	1	0	78
2007	5	45	23	0	0	0	73
2008	2	40	30	0	0	1	73
2009	1	39	23	0	1	0	64
2010	4	38	18	0	0	0	60
2011	0	43	29	2	1	0	75
2012	2	42	18	2	0	0	64
2013	0	35	22	0	1	0	58
2014	0	31	18	0	1	0	50
2015	1	39	27	1	0	0	68
2016	1	39	23	0	1	0	64

**Table 4:** Descriptive statistics of rulings addressing CCs.

	**Category**	**Cases unfounded**		**Cases founded**		**Total**	***p*-value**
		***n*** = **644**		***n*** = **379**			
Animal species	Horses	76	57%	58	43%	134	0.352
	Dogs	374	63%	219	37%	593	
	Cats	138	63%	80	37%	218	
	Rodents	25	68%	12	32%	37	
	Livestock	6	60%	4	40%	10	
	Birds	19	79%	5	21%	24	
	Others	6	86%	1	14%	7	
Animal sex	Male	129	63%	75	37%	204	0.636
	Female	169	61%	108	39%	277	
	Sex unknown	338	64%	188	36%	526	
	Not applicable	3	60%	2	40%	5	
	Both sexes	5	45%	6	55%	11	
Age of animal	Younger than 1 year	42	71%	17	29%	59	0.083
	1–5 years	138	64%	79	36%	217	
	5–10 years	163	69%	73	31%	236	
	10–15 years	95	57%	73	43%	168	
	Older than 15 years	27	69%	12	31%	39	
	Unknown	174	60%	118	40%	292	
	Not applicable	2	50%	2	50%	4	
	Different ages	3	38%	5	63%	8	
Status	Deceased	384	63%	226	37%	610	0.945
	Not Deceased	218	63%	130	37%	348	
	Unknown	38	63%	22	37%	60	
	Not applicable	4	80%	1	20%	5	
Reporting	Reporting important	365	59%	257	41%	622	<0.001
	Reporting not important	279	70%	122	30%	401	
Dog breed	Small	92	63%	55	37%	147	0.777
	Medium	57	67%	28	33%	85	
	Large	171	64%	97	36%	268	
	Weight Unknown	324	62%	199	38%	523	
Summary Complaint	Mistakes veterinary care	383	71%	158	29%	541	0.001
	Communication	153	72%	59	28%	212	
	Other	1	17%	5	83%	6	
	Compliance/general interest	107	41%	157	59%	264	

### Descriptive analysis rulings veterinary appeal council

A total of 177 appeals were submitted during the analyzed period (Table 5). In the majority of these cases, the VAC came to a similar verdict as the VDC (70%). In 18% of the cases, the verdict was lower compared to the VDC and higher in the remaining 12%. Owners, veterinarians, and the CS could appeal, where owners appealed in 53% of the cases and veterinarians appealed in 42%. The variables appellant, type of appeal complaint, and influence of reporting proved to be of statistically significant influence (Table 5).

**Table 5:** Descriptive statistics for the appeal rulings.

**Variables**	**Category**	**Measure equal VTC (*n* = 123)**	**Measure lower VTC (*n* = 32)**	**Measure higher VTC (*n* = 22)**	***n* = 100%**	***p*-value**
Appellant	Owner Veterinarian Civil servant	80 (85.1%) 37 (49.3%) 6 (75%)	7 (7.4%) 24 (32%) 1 (12,5%)	7 (7.4%) 14 (18.7%) 1 (12.5%)	94 75 8	<0.001
Type of Complaint appeal	An identical precedent Procedural grounds Different opinions Technical discussion Other Partially founded	88 (75.2%) 2 (66.7%) 17 (73.9%) 5 (26.3%) 9 (75%) 2 (69.5%)	20 (17.1%) 0 (0.0%) 3 (13%) 6 (31.6%) 2 (16.7%) 1 (33.3%)	9 (7.7%) 1 (33.3%) 3 (13%) 8 (42.1%) 1 (8.3%) 0 (0.0%)	117 3 23 19 12 3	<0.007
Influence reporting	Not important Important	81 (77.1%) 42 (58.3%)	19 (18.1%) 13 (18.1%)	5 (4.8%) 17 (23.6%)	105 72	<0.001

## Discussion

This study is an analysis of 15 years of rulings made by the VDC and VAC. The goal of these rulings is 2-folds: to improve veterinary care and to serve as an objective platform for complaints (2, 3). However, the majority of cases concerned CCs from companion animal owners. Only around 12% of the cases concerned livestock animals, and the majority of the cases were filed by the CS. These CS cases mostly addressed compliance issues and if the veterinarians did not fulfill their duty of care, they are at great risk of being fined, or temporally suspended as a veterinarian. Based on the outcome of the CS cases, we concluded that if it concerns compliance issues, the system works. However, as the number of cases only increased during this period, either the CS became more active in controlling or a learning effect is absent. Although it was not the intent of the Dutch government to create a platform, especially for CCs (2), the vast majority of cases concerned CCs. CCs have a huge effect on the well-being of veterinarians and co-workers (4–6), and the results of this study can be used to improve the level of veterinary care. The impact of the CCs itself was neither the subject of this study nor can we draw any conclusion about what the impact is on the caregiver. This needs to be done in a different setup. But the analysis of the rulings demonstrated that reporting (keeping a proper record of the patient chart), communication, and avoiding veterinary mistakes have a clear influence on the possible outcome of a CC. In this study, there was no apparent significant role observed whether it is a bird, horse, young or old animal and whether the animal died or not played. In several cases, data on age and breed was missing and this may have played a role as rulings with missing data were not analyzed for that category. However, in contrast with CS cases, there was a time effect. During the analyzed period, the number of CC cases and the number of unfound cases decreased. This could be the result of a learning curve: veterinarians learn from their mistakes. During the last part of the analyzed period, every month, disciplinary cases were discussed in the national veterinary journal “Tijdschrift voor Diergeneeskunde.” However, it could also be, as these rulings were made public, the owners only submitted CCs if they assumed that it was a justified case. Nonetheless, the conclusions that can be drawn from this study are very helpful. CCs, like in other countries, play a major role in job satisfaction and the number of newly graduated veterinarians that stop within 5 years after graduation in the Netherlands is almost 17% (13). Hence, job satisfaction is a very important issue to address, and avoiding a CC does help.

Although it should be possible to complain as it may also improve our level of care (6), the detrimental effect on job satisfaction and mental health (4, 5) justified a critical analysis of the rulings.

Looking at the performed analysis, there are, in light of the effect of a CC, some recommendations. In principle, every CC is taken into consideration by the VDC. The legal system does not provide an option to treat complaints of a less heavy character, and/or rooted in the owner's frustration, through a different complaint route. The “unfound” ruling can only be made after a full disciplinary procedure, which plays a major role in the veterinarian's well-being and job satisfaction (4–6). It seems safe to conclude that the current method of working could be adapted.

This study has limitations. The actual impact of a CC on the veterinarian could not be studied. It is not possible to establish the exact amount of learning effect. It would have been very interesting to compare the outcome of this study with different veterinary disciplinary systems in other countries. There is, one recent Californian study providing insights from the veterinary disciplinary system, but this study addresses different issues (4). But all of these questions will be subject to additional studies.

## Conclusion

It is concluded that compliance, performing its duty of care, proper record keeping, good communication, and avoiding veterinary mistakes can reduce the number of CS complaints and CCs. Additional studies are needed to investigate the impact and learning effect of CCs on veterinarians.

## Data availability statement

The raw data supporting the conclusions of this article will be made available by the authors, without undue reservation.

## Author contributions

IB and PM were responsible for the conception of the study, acquisition of the data, and writing–review and editing of the manuscript. MR and PM were responsible for the analysis, interpretation, and the statistical analysis. AJ, FM, and JH were responsible for writing–review and editing of the manuscript. All authors contributed to the article and approved the submitted version.

## Conflict of interest

The authors declare that the research was conducted in the absence of any commercial or financial relationships that could be construed as a potential conflict of interest.

## Publisher's note

All claims expressed in this article are solely those of the authors and do not necessarily represent those of their affiliated organizations, or those of the publisher, the editors and the reviewers. Any product that may be evaluated in this article, or claim that may be made by its manufacturer, is not guaranteed or endorsed by the publisher.

## Footnotes

^1^Microsoft Excel 2022.

^2^SPSS version 28 for statistical analyses.

## Abbreviations

CC: Client Complaint
CS: Civil Servant
MvT: Memorial of Explanation
VDC: Veterinary Disciplinary Council
VAC: Veterinary Disciplinary Appeal Council
WUD: Dutch Veterinary Act 1990.
