# Supplementary material for: 15 years of facts and figures on veterinary disciplinary measures in the Netherlands
Source: Front Vet Sci. 2022 Nov 11;9:987797. doi: 10.3389/fvets.2022.987797 (PMC9691658; doi:10.3389/fvets.2022.987797)
Supplement: Supplementary file 1 [file Data_Sheet_1.docx]

Appendix I Parameters VDC

| **Category** | **Value** | **Number** |
| --- | --- | --- |
| Animal species | Horses | 1 |
| Animal species | Dogs | 2 |
| Animal species | Cats | 3 |
| Animal species | Rodents | 4 |
| Animal species | Livestock | 5 |
| Animal species | Poultry | 6 |
| Animal species | Birds | 7 |
| Animal species | Others | 8 |
| Main animal category | Companion animals | 1 |
| Main animal category | Horses | 2 |
| Main animal category | Livestock | 3 |
| Dog size | Size unknown | 1 |
| Dog size | Small <10 kg | 2 |
| Dog size | Medium 10-20kg | 3 |
| Dog size | Large > 25kg | 4 |
| Animal sex | Sex unknown | 1 |
| Animal sex | Male | 2 |
| Animal sex | Female | 3 |
| Animal sex | Not applicable | 4 |
| Animal sex | Both sexes (male and female) | 5 |
| Animal age | Unknown | 1 |
| Animal age | Not applicable | 2 |
| Animal age | Younger than 1 year | 3 |
| Animal age | 1 - 5 years | 4 |
| Animal age | 5 - 10 years | 5 |
| Animal age | 10 - 15 years | 6 |
| Animal age | Older than 15 years | 7 |
| Animal age | Various ages | 8 |
| Animal deceased | Deceased | 1 |
| Animal deceased | Not deceased | 2 |
| Animal deceased | Unknown | 3 |
| Animal deceased | Not applicable | 4 |
| Complainer | Owner | 1 |
| Complainer | Civil servant | 2 |
| Summary complaint | Complications due to treatment | 1 (1) |
| Summary complaint | Communication | 2 (2) |
| Summary complaint | Communication / Duty of care | 3 (2) |
| Summary complaint | Different opinion of owner | 4 (2) |
| Summary complaint | Ethical reasons | 5 (2) |
| Summary complaint | Euthanasia complications | 6 (1) |
| Summary complaint | Euthanasia without permission | 7 (1) |
| Summary complaint | Euthanasia request outside office hours | 8 (1) |
| Summary complaint | Financial reasons | 9 (2) |
| Summary complaint | Confidentiality | 10 (2) |
| Summary complaint | Cause of death unknown | 11 (1) |
| Summary complaint | Not enough diagnostics used | 12 (1) |
| Summary complaint | Not enough examination | 13 (1) |
| Summary complaint | Other reasons | 14 (1) |
| Summary complaint | Death due to treatment | 15 (1) |
| Summary complaint | Death after surgery | 16 (1) |
| Summary complaint | Death after sedation | 17 (1) |
| Summary complaint | Wrong treatment | 18 (1) |
| Summary complaint | Wrong diagnosis | 19 (1) |
| Summary complaint | Refuses to offer help | 20 (1) |
| Summary complaint | Wrong diagnose and Treatment | 21 (1) |
| Summary complaint | Legal issues (WUD) | 22 (3) |
| Summary complaint | Carefulness of acting | 23 (4) |
| Outcome/measure | Dismissed or inadmissible | 1 |
| Outcome/measure | Unfounded | 2 |
| Outcome/measure | Founded without measure | 3 |
| Outcome/measure | Warning | 3 |
| Outcome/measure | Reprimand | 3 |
| Outcome/measure | Fine conditional or unconditional | 4 |
| Outcome/measure | Fine and/or (un)conditional suspension | 5 |
| Outcome/measure | Partial or complete suspension | 6 |
| Outcome ruling | Unfounded | 1 |
| Outcome ruling | Founded | 2 |
| Influence reporting | Reporting important | 1 |
| Influence reporting | Reporting not important | 2 |

Appendix II Additional parameters VAC

| **Category** | **Value** | **Number** |
| --- | --- | --- |
| Summary complaint | Content VTC unknown | 24 |
| Appellant | Owner | 1 |
| Appellant | Veterinarian | 2 |
| Appellant | Civil servant | 3 |
| Summary appeal | An identical precedent | 1 |
| Summary appeal | Procedural grounds | 2 |
| Summary appeal | Different opinions | 3 |
| Summary appeal | Technical discussion | 4 |
| Summary appeal | Other: Challenge court, Inadmissible | 5 |
| Summary appeal | Partially founded | 6 |
| Outcome VBC | Rejection | 1 |
| Outcome VBC | Inadmissible | 2 |
| Outcome VBC | Founded | 3 |
| Outcome VBC | Unfounded | 4 |
| Outcome VBC | Destroy judgement | 5 |
| Outcome VBC | Reject revision VBC case | 6 |
| Comparison VTC | Measure equal VTC | 1 |
| Comparison VTC | Measure lower VTC | 2 |
| Comparison VTC | Measure higher VTC | 3 |
